# Clinical outcomes of osteonecrosis of the femoral head after autologous bone marrow stem cell implantation: a meta-analysis of seven case-control studies

**DOI:** 10.6061/clinics/2016(02)10

**Published:** 2016-02

**Authors:** Heng-feng Yuan, Jing Zhang, Chang-an Guo, Zuo-qin Yan

**Affiliations:** Fudan University, Zhongshan Hospital, Department of Orthopedics, Shanghai, China

**Keywords:** Osteonecrosis, Femoral Head, Bone Marrow, Meta-Analysis

## Abstract

The purpose of this study was to evaluate the clinical outcomes of osteonecrosis of the femoral head after autologous bone marrow stem cell implantation. We searched the PubMed, Embase and Web of Science databases and included all case-control trials that reported on the clinical outcomes of osteonecrosis progression, incidence of total hip arthroplasty and improvement in Harris hip scores. Overall, seven case-control trials were included. Compared with the controls, patients treated with the bone marrow stem cells implantation treatment showed improved clinical outcomes with delayed osteonecrosis progression (odds ratio = 0.17, 95% CI: 0.09 – 0.32; *p*<0.001), a lower total hip arthroplasty incidence (odds ratio = 0.30, 95% CI: 0.12 - 0.72; *p*<0.01) and increased Harris hip scores (mean difference = 4.76, 95% CI: 1.24 – 8.28; p<0.01). The heterogeneity, publication bias, and sensitivity analyses showed no statistical difference significant differences between studies. Thus, our study suggests that autologous bone marrow stem cells implantation has a good therapeutic effect on osteonecrosis of the femoral, resulting in beneficial clinical outcomes. However, trials with larger sample sizes are needed to confirm these findings.

## INTRODUCTION

Osteonecrosis of the femoral head (ONFH) is a debilitating and painful disease associated with multiple risk factors, such as trauma, corticosteroid administration, alcohol abuse, organ transplantation, and some inflammatory or autoimmune diseases 1,2. Without effective early treatment, this type of osteonecrosis can develop into femoral head collapse with subsequent hip joint destruction and patients may eventually require total hip arthroplasty (THA) to restore joint function 3. As ONFH mainly affects young and middle-aged adults and THA cannot be expected to last the patient's lifetime, hip-preserving treatments are especially important for these patients 4,5.

Recent reports 6,7 have shown that bone marrow stem cell (BMSC) implantation into the necrotic lesion of the femoral head is a promising cellular-based therapy. The pathogenesis of ONFH involves both vascular and bone pathology with altered bone remodeling 8,9,10. BMSC function to promote angiogenesis and osteogenesis, although the number and activity of these cells are decreased in the femoral head of patients with ONFH 11,12. Hence, autologous BMSC implantation could be useful for the treatment of ONFH.

To date, several original trials 13,14,15 have reported the use of BMSC for ONFH treatment. However, the clinical outcomes were not conclusive. These inconclusive results could be attributed to the small sample size in each of the reported trials and the low statistical power of the individual studies. Therefore, in this study, we performed a meta-analysis to investigate the clinical outcomes of ONFH after BMSC implantation.

## METHODS

### Search strategy

This meta-analysis was performed in accordance with the PRISMA guidelines 16. A systematic literature search of PubMed, Embase and Web of Science databases through March 10, 2015 was conducted. Combinations of the terms “bone marrow stem cell” or “bone marrow mononuclear cell” or “bone marrow-derived cell”; “osteonecrosis” or “avascular necrosis”; and “femoral head” or “femur” were used without restricting the language or publication date. Relevant studies were retrieved accordingly. In addition, we also checked the references of the articles to identify other relevant publications.

### Selection criteria

Two authors independently reviewed the titles and abstracts of potentially relevant studies. The inclusion criteria consisted of the following: (a) BMSC (including bone marrow cells or bone marrow mononuclear cells) implantation used to treat ONFH patients; (b) trials including a control group without BMSC implantation; (c) no less than one year of follow-up time; and (d) studies reporting at least one of the following clinical outcomes: ONFH progression, THA incidence and improvement of Harris hip scores (HHS). If the clinical outcomes were reported more than once by the same research group, we included the report with the longest follow-up time and the largest number of patients.

### Data extraction and quality assessment

For each trial, we extracted the following items: (a) the surname of the first author; (b) the year of publication; (c) the BMSC treatment method; (d) the number of patients; (e) the age of the patients; (f) the follow-up time; (g) the radiological ONFH progression at the last follow-up; (h) the THA incidence at the last follow-up; and (i) the changes in HHS at the last follow-up.

Quality assessments were performed independently by two authors, and any disagreements between authors were resolved by discussion. In addition, the evidence level of each study was determined according to the Cochrane handbook for systematic reviews of interventions 17.

### Statistical analysis

Analyses were performed using the software Review Manager, version 5.0 (RevMan, The Cochrane Collaboration, Oxford, UK) and STATA package v.11.0 (Stata Corporation, College Station, TX, USA). Differences between patients receiving BMSC treatment and controls were expressed with the pooled odds ratio (OR) or mean difference (MD) and 95% confidence interval (CI). The fixed effect model was used for analysis. However, if significant heterogeneity existed between trials (a *p*-value of Q test <0.10 or/and I^2^>50%), the random effect model was used instead. Publication bias was evaluated with funnel plots using Egger's regression model. Sensitivity analyses were performed based on the “1-study removed” analyses. A *p*-value <0.05 was considered statistically significant.

## RESULTS

### Trials included in the meta-analysis

**Figure 1-:**
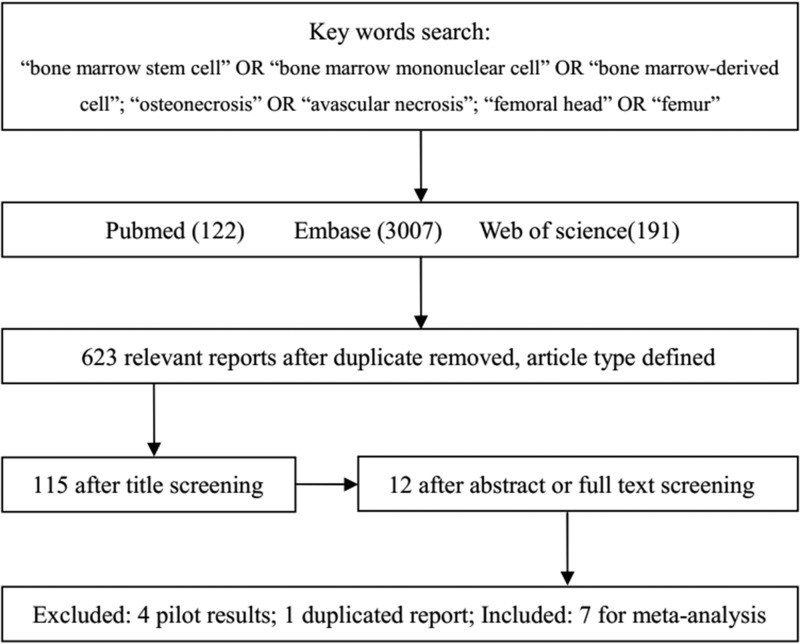
Flowchart of the selection process for the meta-analysis.

A total of 3,320 articles were retrieved from the PubMed, Embase and Web of Science databases, of which 12 studies potentially met the identified criteria (Figure 1). Subsequently, 4 studies were excluded because they reported on early/pilot follow-up results; another study was excluded because the results were published twice in the same year in two different languages. In the end, 7 trials 13-15, were included in the meta-analysis and their detailed characteristics are displayed in Table 1.

### Clinical outcomes after BMSC implantation

**Figure 2-:**
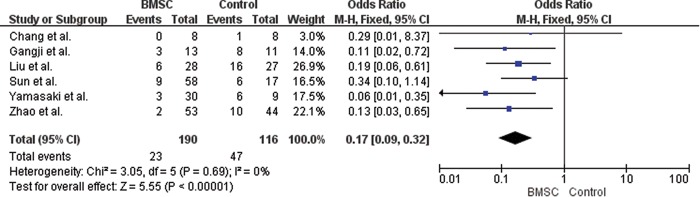
Forest plot for the progression of osteonecrosis of the femoral head after bone marrow stem cell implantation.

**Figure 3-:**
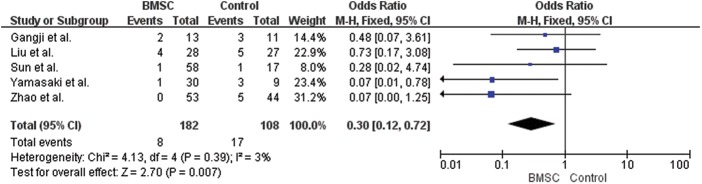
Forest plot for the arthroplasty incidence after bone marrow stem cell implantation.

**Figure 4-:**
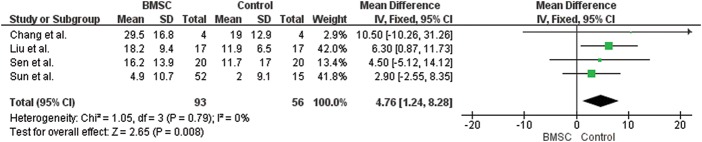
Forest plot for the HHS improvement after bone marrow stem cell implantation.

Among the 7 trials, 216 hips were treated with BMSC implantation, compared with 141 controls. No heterogeneities were observed between the two groups (ONFH progression: I^2^ = 0%, *p*=0.69; THA incidence: I^2^ = 3%, *p*=0.39; HHS: I^2^=0%, *p*=0.79, respectively); hence, the fixed effect models were used. The overall meta-analysis including the 6 trials indicated that the pooled OR for ONFH progression was 0.17 (95% CI: 0.09 - 0.32; *p*<0.001) (Figure 2). The pooled OR for THA incidence was 0.30 (95% CI: 0.12 - 0.72; *p*<0.01) (Figure 3). The analysis of the improvement of HHS revealed that the pooled MD was 4.76 (95% CI: 1.24 – 8.28; *p*< 0.01) (Figure 4). These results indicated that patients who underwent BMSC implantation showed significantly better clinical outcomes than the control group.

### Publication bias and sensitivity analysis

**Figure 5-:**
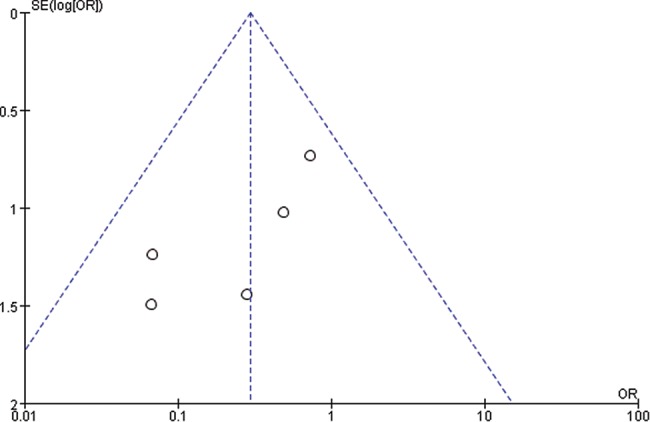
Funnel plot for the detection of publication bias (comparison of arthroplasty incidence).

No significant publication bias (*p*>0.05) was found in any of the pooled comparisons based on Egger's test for funnel plot asymmetry (Figure 5 for detection of THA incidence comparison). In addition, the sensitivity analysis showed no changes in the results when any trial was excluded.

## DISCUSSION

BMSC implantation is an effective method for the treatment of ONFH. These cells can be isolated from individual patients without heterologous hazards or ethical questions 22 and no direct complications have ever been reported following this type of therapy 6. BMSC show multi-potential capacities to differentiate into osteoblasts, endothelial cell progenitors and hemangioblasts, which function to repair the necrosis region of the femoral head 22,23. In addition, reports 24,25 have shown that BMSC also release a variety of growth factors, such as VEGF-A, to facilitate bone regeneration. Our previous research 25 demonstrated that BMSC can not only survive but also expand in the necrotic area for up to 12 weeks after implantation.

Although randomized, controlled trials would provide the strongest evidence, few randomized trials have reported on the application of BMSC implantation for the treatment of ONFH. The next-best alternative is comparative case-control trials; however, these reports have shown conflicting clinical outcomes, which may be due to the small sample sizes and low statistical power of the studies. Hence, in this study, we performed a comprehensive and systematic analysis of the data from 6 independent case-control trials. The results showed that BMSC implantation delayed the progression of ONFH and decreased the need for THA. In addition, BMSC implantation may also improve hip function, as demonstrated by the increased HHS in treated patients.

There are some limitations in our study that should be considered. First, although seven independent studies were included, the total number of cases and controls in the meta-analysis was relatively small. Second, the methods of BMSC implantation reported in these trials were not completely uniform, which could have influenced the validity of our analysis. Third, we failed to analyze the changes in the volume of the necrotic lesion as insufficient data were reported in the included studies.

In summary, our study found that BMSC implantation resulted in better clinical outcomes of ONFH, delayed ONFH progression, decreased THA incidence and improved HHS. However, further studies with larger sample sizes are needed to confirm these results.

## AUTHOR CONTRIBUTIONS

Yan Z developed the idea for the study and drafted the manuscript. Yuan H and Zhang J were responsible for conducting the literature search, data collection and study quality assessment. Yuan H and Guo C analyzed and interpreted the data. All the authors read and approved the final version of the manuscript.

## Figures and Tables

**Table 1:** Characteristics of the 357 hips identified from the literature.

Authors	Year	Treatment	No. of patients (hips)	Age (mean)	Follow-up (months)
			*Case/Control*	*Case/Control*	*Case/Control*
Sun et al.	2008	BMSC	45(58)/13(17)	37.5/37.3	12/12
Yamasaki et al	2010	BMMC+IP-CHA	22(30)/8(9)	41/49	29/31
Chang et al	2010	BMSC	4(8)/4(8)	-/-	23.5/23.5
Gangji et al.	2011	BMSC	13/11	42.2/45.7	60/60
Zhao et al.	2012	BMSC	50(53)/43(44)	32.7/33.8	60/60
Sen et al.	2012	BMMC	20(26)/20(25)	-/-	24/24
Liu et al.	2013	BMMC+NH/PA	17(28)/17(27)	38.0/38.1	26.7/24.9

**Note:** BMSC: bone marrow stem cells, BMMC: bone marrow mononuclear stem cells, IP-CHA, NH/PA: both are implant materials.
